# Atypical Seropositive Striated Muscle Antibody Myasthenia Gravis Presentation With Metastatic B1 Thymoma: A Rare Case

**DOI:** 10.7759/cureus.35221

**Published:** 2023-02-20

**Authors:** Johnny S Randhawa, Lauren S Kim, Cesar Aguilar, Alexander T Phan, Hadi Abou-El-Hassan, Lisa Herring Sovory

**Affiliations:** 1 Internal Medicine, Arrowhead Regional Medical Center, Colton, USA; 2 Internal Medicine, California University of Science and Medicine, Colton, USA; 3 Neurology, Arrowhead Regional Medical Center, Colton, USA; 4 Neurology, California University of Science and Medicine, Colton, USA

**Keywords:** striational antibodies, b1 thymoma, myasthenia gravis (mg), anterior mediastinal mass, thymoma

## Abstract

The association between myasthenia gravis (MG) and thymomas is well-documented. Thymomas are rare epithelial cell tumors that arise from the thymus gland and occur in the mediastinum. Myasthenia gravis is a neuromuscular disorder that causes skeletal muscle weakness due to the presence of anti-acetylcholinesterase antibodies. Roughly 60% of thymomas are associated with MG, while only 10% of MG patients have thymomas. We present an atypical presentation of myasthenia gravis with an associated unusual metastatic thymoma. This case is of a young, previously healthy 26-year-old male with no previous medical history who presented with non-specific symptoms of fatigue, diarrhea, abdominal pain, back pain, blurry vision, and unintended weight loss. He underwent treatment with intravenous immunoglobulins (IVIG), had two surgical resections of the thymoma, and ultimately received radiotherapy. Based on our experience with this case, diagnosing myasthenia gravis by testing for specific muscle antibodies for patients with ptosis in the setting of non-specific complaints, including fatigue, vomiting, diarrhea, and abdominal or back pain, should be considered. Routine imaging should follow with a chest computed tomography to screen for thymomas if the specific anti-titin and anti-ryanodine receptor (anti-RyR) muscle antibodies are positive and myasthenia gravis is suspected. If a thymoma is confirmed, it is best to confirm; and mass characterizes with chest magnetic resonance imaging. A treatment approach of IVIG followed by surgical resection and possible debulking if the lesion is deemed metastatic could also be considered thereafter, especially in young patients with few comorbidities. Treatment with Pyridostigmine 30 mg twice daily for 25 days post-surgically and radiation for treatment of any remaining unresectable tumor should also be considered.

## Introduction

The association between myasthenia gravis (MG) and thymomas, a mediastinal tumor, is well-documented. Myasthenia gravis is a neuromuscular autoimmune disorder that causes skeletal muscle weakness due to the presence of antibodies, including but not limited to anti-acetylcholinesterase, Musk, LRP4, and seronegative antibodies [1]. Mediastinal tumors are uncommon and represent 3% of tumors seen within the thoracic cavity, including thymoma, lymphoma, pheochromocytoma, germ cell tumors, and parathyroid lesions; the most common of these is a thymoma [2-3]. Thymomas are rare epithelial cell tumors that arise from the thymus gland and occur in the mediastinum [4]. Roughly 60% of thymomas are associated with MG, while only 10% of MG patients have thymomas [4]. There are various subtypes of thymomas that are classified by the World Health Organization (WHO) as well as the Masaoka system.

The development of myasthenia gravis as a paraneoplastic syndrome of cortical thymoma is exceedingly rare. The pathophysiology of this development is thought to be due to cancer’s retained ability to secrete mature naive T cells into the periphery. These aberrant T cells may play a role in the development of autoantibodies which may contribute to the pathophysiology of myasthenia gravis [4].

80%-85% of patients with MG will be positive for acetylcholine receptor antibodies (anti-AChR), irrespective of the presence of a thymoma [5]. Myasthenia gravis is associated with seropositivity for several auto-antibodies, some of which predict disease severity and prognosis. Early-onset (age <40 years) MG patients with generalized weakness and a thymoma tend to have the highest concentrations of AChR binding antibodies; however, the anti-AChR titer does not predict disease severity [6].

The pathogenicity of anti-AChR antibodies in MG is well supported with evidence; however, non-AChR antibodies, such as anti-titin and ryanodine receptor antibodies (anti-RyR), that react with striated muscle antigens may also be present in up to 95% of MG patients with thymomas [7]. Thus, antibodies reactive to striated muscle may aid in the diagnosis of thymomatous MG and may be indicative of thymic disease. They are particularly helpful as a thymoma marker in patients with anti-AChR-positive MG who present before the age of 40. Anti-striated muscle antibodies can be directed against several different parts of the striated muscle, including titin, myosin, actin, and ryanodine receptors. Anti-titin antibodies are present in 70%-90% of MG patients with thymoma and may be associated with a more severe disease course [7]. Additionally, ryanodine receptor antibodies are also closely associated with thymomatous MG patients. The sensitivity for thymomatous MG patients is highest when testing for both these antibodies together [7].

Thymomas are graded and staged by the World Health Organization histological classification and Masaoka system, respectively. Thymomas can be classified, per the fifth edition of WHO Classification of Thoracic Tumours, into type A, type AB, and type B and further subdivided into B1, B2, and B3. They are based predominantly on histologic features but also immunohistochemistry [2]. The Masaoka Koga staging system for thymic malignancies also plays a critical role in prognostication and is primarily based on local invasion of the primary tumor and degree of nodal involvement [3,4,8]. Another factor that should not be overlooked when determining thymoma prognosis is tumor resectability [6]. Through considerations of the WHO thymoma classification, Masaoka Koga system, and tumor resectability, prognostication may be most accurate, as this process likely requires a multifactorial approach.

The typical first-line treatment for MG patients with thymoma is surgical removal, which is typically curative [4]. Malignant invasive thymoma in the setting of myasthenia gravis is rare and has only been previously documented in several young patients [8].

## Case presentation

A 26-year-old Caucasian male with a past medical history of recently diagnosed myasthenia gravis with thymoma one month before admission presented to the emergency department with symptoms of fatigue, intermittent blurry vision, vomiting, diarrhea, abdominal pain, back pain, and unintentional weight loss. He had presented to another hospital six months prior with similar symptoms. He denied diplopia, eyelid drooping, and muscle weakness. During his hospitalization at the outside facility six months prior, a biopsy of his mediastinal mass led to a definitive diagnosis of a WHO B1 thymoma with metastasis to the pleura. The mass at the time was determined to be unresectable due to a high disease burden and metastasis. Additionally, a diagnosis of myasthenia gravis was also confirmed with seropositivity for anti-AChR binding, anti-AChR blocking, and anti-striated muscle antibodies. He was discharged from the outside hospital on 10 mg oral prednisone and 20 mg oral pyridostigmine once daily, which the patient took for two weeks; however, he then discontinued the medications because they failed to improve his symptoms.

**Figure 1:**
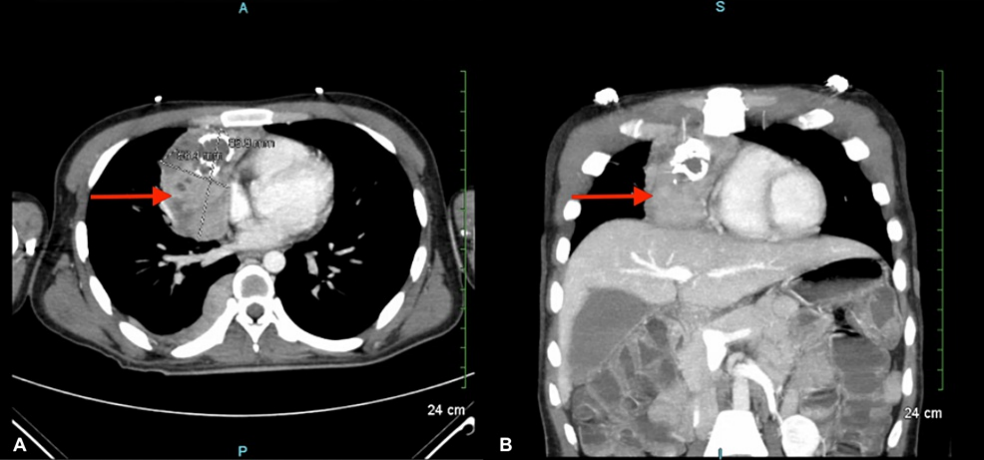
A: Axial section of chest CT with IV contrast showing a large right mediastinal mass and right posterior pleural thickening. B: Coronal section of chest CT with IV contrast showing a large right mediastinal mass with irregular prior to thymectomy and debulking. CT: computed tomography, IV: intravenous

The patient presented to our hospital due to a recurrence of his symptoms. On arrival, the patient’s vitals were as follows: blood pressure of 99/67 mmHg, heart rate of 155, the temperature of 98 F, respiratory rate of 20, and oxygen saturation of 99% on room air. On neurological exam, the patient was alert and oriented to person, place, and time with a GCS of 15. Cranial nerves II-XII were intact, and the patient had no sensory deficits in the upper or lower extremities. The patient had generalized motor weakness throughout. Otherwise, patellar and Achilles reflexes were +4 bilaterally. An ice pack test was negative. He was then placed on observation for cardiac and respiratory monitoring, using telemetry and negative inspiratory force (NIF) measurements, respectively, for three weeks. He was given intravenous crystalloid fluids for his tachycardia and relative hypotension. A chest computed tomography (CT) scan of the chest showed a large right lung mass with irregular right posterior thoracic pleural thickening (Figures 1A, 1B). A cardiothoracic surgeon was consulted, and a mass resection was scheduled for 12 days after admission. The patient received 400 mg IVIG daily for five days pre-operatively. Additionally, the patient's vital capacity and negative inspiratory force values were continuously monitored to assess for any acute changes in respiration (Table 1). He did not require any respiratory support and was stable on room air.

**Table 1:** SpO2, vital capacity, and negative inspiratory pressure monitored over the course of three weeks time. This table demonstrates slow improvement in respiratory values over the course of three weeks. SpO2: oxygen saturation, L: Liters, RA: room air, ml: milliliter, cm: centimeter, H2O: water

	Week 1	Week 2	Week 3
SpO2	98%-100% on RA	97%-98% on RA	97%-98% on RA
Vital Capacity (L)	1.1 - 2.4	0.65 - 2.9	0.88 - 1.8
Negative Inspiratory Force (cm H2O)	(-40) - (-22)	(-44) - (-20)	(-60) - (-28)

**Figure 2:**
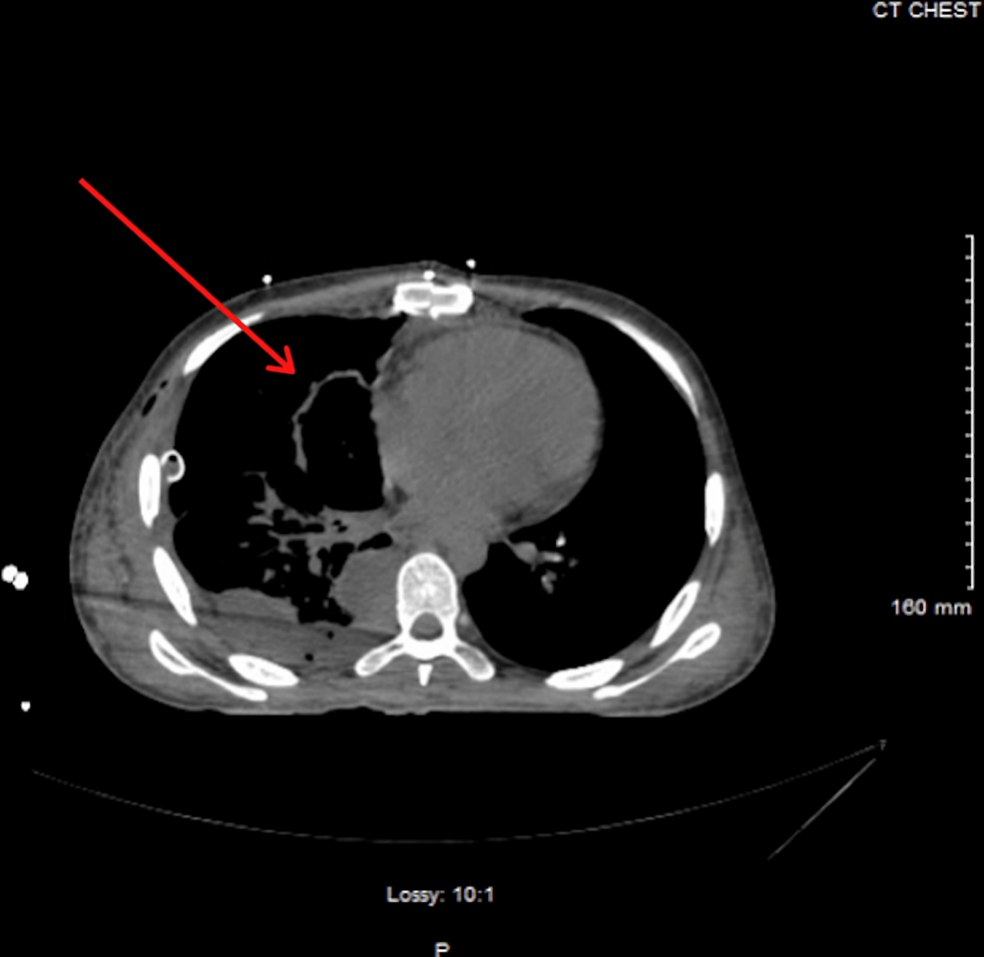
Axial section of a chest computed tomography without intravenous contrast showing interval resection of the mediastinal mass post-surgically.

**Figure 3:**
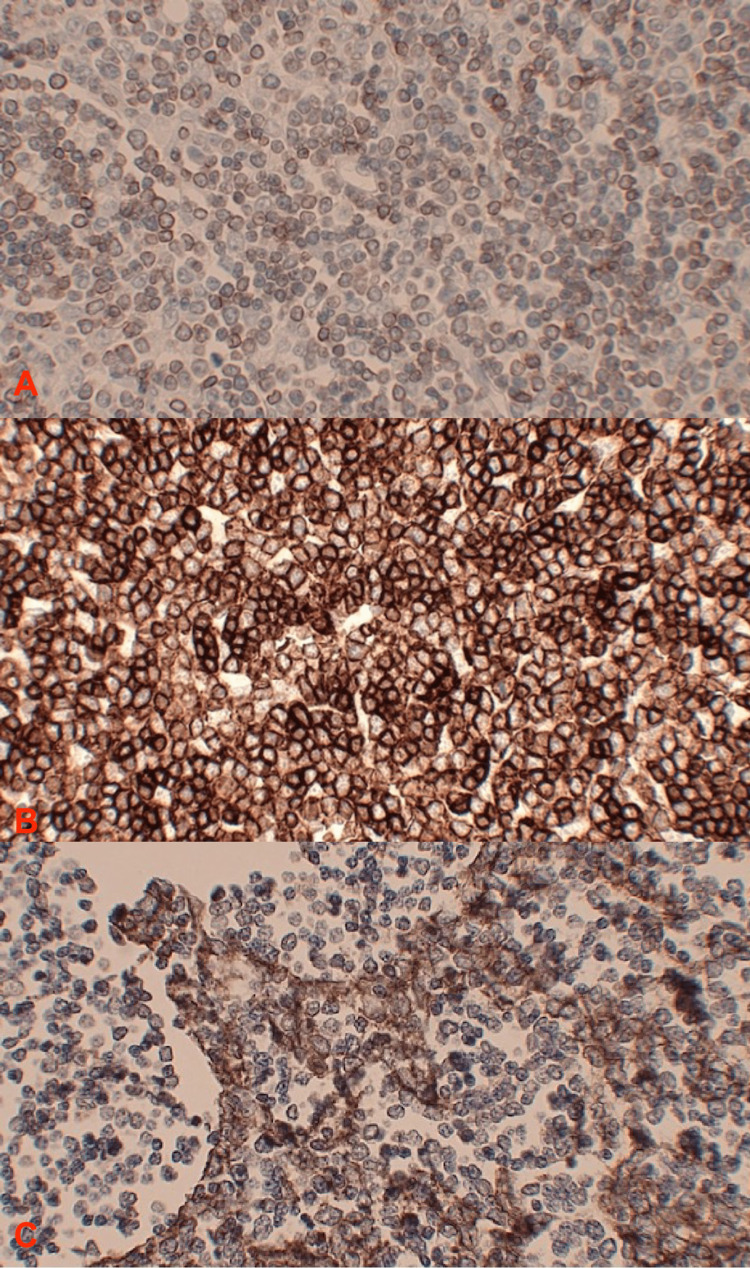
A: CD3 staining of resected thymoma (Immunohistochemistry, low power), B: Leukocyte common antigen staining of resected thymoma (Immunohistochemistry, high power), C: Pancytokeratin staining of resected thymoma (Immunohistochemistry, high power) CD: A cluster of differentiation

On hospital day 13, the patient underwent a thymectomy with multiple biopsies, where a large right anterior mediastinal mass was resected; postoperative chest imaging can be seen in Figure 2. Biopsies from the right pleural cavity, thymus, and right pericardial mass were collected, and pathological results can be seen in Figures 3A-3C. The surgery was uncomplicated, and results from the final pathology report can be seen in Table 2. There were no major complications, and the patient tolerated the surgery well. On hospital day 34, the patient underwent a second thoracotomy due to extensive disease burden and debulking of his metastatic thymoma, where approximately 70% of adhesions from the lung to the chest wall were removed. Per the surgical procedure note, an extensive tumor burden extended posteriorly to the spine and inferiorly to the diaphragm, which was completely adherent to the pleura. The tumor was noted to be vascular, and while debulking, the surgery was complicated by an intrathoracic hemorrhage with an estimated blood loss of 2.5 liters. The patient received six units of packed red blood cells, four units of fresh frozen plasma, and one unit of platelets during the procedure. The patient additionally underwent a partial pleurectomy to remove the tumor from the pleural space. On hospital day 38, the patient was started on Pyridostigmine 30 mg twice daily, which was continued for 25 days, at which point the patient was deemed stable for discharge. The patient was also started on radiotherapy in the inpatient setting, with a plan for a total of 25 fractions to be completed on an outpatient basis.

**Table 2:** Final pathology results. WHO: World Health Organization

A. Pleural cavity, right: Necrotic and infarcted tissue in part surrounded by fibroadipose tissue with inflammatory infiltrate. No viable tumor cells are present.
B. Thymus: Thymoma, WHO type B1. Residual thymic tissue with follicular lymphoid hyperplasia.
C. Right pericardial mass: Thymoma, WHO type B1.

 

## Discussion

Antibodies against striated muscle (striational antibodies)

In the thymomatous subtype of MG, non-AChR auto-antibodies reactive to striated muscle antigens occur up to 95% of the time [7]. Given the relationship between thymomatous MG and striatal antibodies, the presence of these antibodies may be diagnostic of thymomatous MG and may reflect thymic pathology [9]. As a marker of thymoma, striatal antibodies are most useful in patients who develop anti-AChR-positive MG before age 40 [7], as seen with our young patient who was positive for anti-skeletal muscle and anti-AChR antibodies. Hence, patients less than 40 years of age who test positive for anti-striated muscle antibodies should be screened for anterior mediastinal masses, specifically thymoma, using a CT scan of the chest, as was done with our patient. In patients who are found to have lesions suspicious of thymoma on imaging, a further biopsy is warranted to help classify the subtype of thymoma, which may help direct management and determine prognosis.

One of the few antigenic targets of striatal antibodies is titin, an intracellular protein [10]. Anti-titin antibodies are present in approximately 70%-90% of MG patients with thymoma [6,11]. MG patients with anti-titin antibodies often experience worse myopathy and more severe muscle weakness than seronegative MG patients [7]. Because the thymus expresses titin, a hyperplastic thymus or thymoma may be the primary site for sensitization of antibodies to titin antigen; however, patients without thymus pathology can also be positive for anti-titin antibodies [12-13]. Overall, as 95% of patients with thymomatous MG have anti-titin antibodies, the titin antibody assay is clinically useful, and its sensitivity is essentially equivalent to that of chest CT [6,11]. Unfortunately, the specificity of titin antibodies for thymoma is low, and approximately 50% of patients with late-onset MG may have these antibodies irrespective of a thymoma present [6,11]. Ryanodine receptor (RyR) antibodies, a different type of antigenic target, are also closely associated with thymomatous MG, and patients usually have pronounced bulbar and respiratory muscle weakness [14-16]. RyR antibodies belong mainly to the IgG1 and IgG3 subclasses, are capable of activating complement and can inhibit calcium release from the sarcoplasmic reticulum in vitro [17-18]. Several reports have also suggested that RyR antibodies impair excitation-contraction coupling in thymoma MG [19]. RyR antibodies are more specific for thymoma but are found in only about 75% of MG patients with thymoma [7]. Thus, the combination of titin and RyR antibody positivity may be more useful, as studies have demonstrated that this combination has approximately 95% sensitivity and 70% specificity for MG thymoma [7].

In summary, myasthenia gravis is an autoimmune condition characterized by positive anti-antibodies, including but not limited to ach, Musk, LRP4, and seronegative antibodies, and may or may not be associated with the development of a thymoma. Once a new diagnosis of MG is made, it is a standard procedure to look for thymic pathology by imaging to screen for a thymoma. It may also be useful to acquire specific muscle striation antibodies tests, including anti-titin and anti-RyR antibodies, as this would guide management strategies and prognostication.

Classification of subtypes of thymomas

Once it has been identified that a patient with myasthenia gravis has a mediastinal mass, a biopsy is warranted to establish the diagnosis and determine the subtype, given that it is a thymoma. Determining the subtype will help further guide management and determine prognosis. Our patient was diagnosed with a WHO classification B1 thymoma, which showed a lymphocyte-rich tumor with healthy cells in the thymus (Tables 3, 4), and more than half of his thymoma was resected [9,20].

**Table 3:** WHO Classification of Thymic Tumors (adapted from Marx A, Willcox N, Leite MI, et al., 2022) WHO: World Health Organization

WHO Classification	Epithelial cells	T-cells	Other desirable features
Type A	Bland spindle and oval thymic epithelial cells	Very few or no immature TdT(+) T-cells	High expression of epithelial markers
Type AB	Bland spindle, oval, or polygonal thymic epithelial cells	Abundance of TdT(+) T-cells, either focal or diffuse	Lobulated tumor with dense TdT staining
Type B1	Dispersed thymic epithelial cells	Densely packed TdT(+) T-cells	Thymic-like architecture with sheets of TdT(+) T-cells and scattered TdT(-) T-cells
Type B2	Numerous neoplastic epithelial cells, often in clusters	Abundant TdT(+) T-cells	Keratin or p40/p63 staining to highlight neoplasitc epithelial cells
Type B3	Atypical polygonal cells arranged in sheets	Scarce TdT(+) T-cells	Lobulated tumor with perivascular spaces and TdT staining showing scare T-cells

**Table 4:** Masaoka Staging System Table of Thymic Tumors (adapted from Detterbeck FC, Nicholson AG, Kondo K, et al., 2011)

Staging	Invasion?	Metastasis?
I	No invasion microscopically; completely encapsulated	Not present
IIa	Microscopic invasion across capsule	Not present
IIb	Macroscopic invasion into surrounding tissues (e.g., thymus and surrounding adipose)	Not present
III	Macroscopic invasion into surrounding organs (e.g., lungs and pericardium)	Not present
IVa	Macroscopic invasion	Metastases to the pleura or pericardium
IVb	Macroscopic invasion	Hematogenous or lymphatic metastases

Treatment

Our patient received five days of IVIG preoperatively to reduce the possibility of a myasthenic crisis during surgery [21]. A literary search reveals that, compared to plasmapheresis, IVIG has equal or improved results perioperatively. Hence, it may be reasonable to initiate IVIG therapy before surgery or resection of a thymoma [22]. Additionally, multiple surgical resections can be performed as tolerated by the patient until the appropriate amount of thymoma has been resected. Our patient received oral Pyridostigmine therapy, which led to an overall improvement in his symptom of bilateral ptosis, hyperreflexia, and mild improvement in his generalized weakness. This adjunctive therapy may also be useful in thymomatous MG [23-24], though more studies would be needed to clarify the most appropriate dosing regimen. Finally, radiation therapy may also be therapeutic for patients with unresectable masses, and the decision regarding dosing should be made by a radiation oncology specialist. In caring for MG patients with symptoms of MG crisis and a malignant thymoma, treatment with intravenous immunoglobulin (IVIG), Pyridostigmine, and surgical removal of the mass should be considered. Additionally, radiation therapy should be considered for any unresected mass.

## Conclusions

In this case report, we discuss a rare manifestation of a large metastatic thymoma with an atypical presentation of myasthenia gravis, which, to our knowledge, has only been described twice before. Routine imaging studies are the standard for mediastinal masses in MG patients. Physicians may also screen for specific antibodies, including anti-titin and anti-RyR, which, if positive, are markers highly associated with thymomatous MG. In our experience, IVIG, subsequent resection of the thymoma, chemotherapy, and radiotherapy, as a multifactorial approach to the management of combined metastatic thymoma and myasthenia gravis, may provide success in managing this rare disease entity. Future studies should evaluate the most optimal treatment regimen and diagnostic work-up, as the current literature is very limited in managing this condition.
